# A neonatal case of non‐eosinophilic esophagitis type eosinophilic gastrointestinal disease diagnosed by rectal biopsy

**DOI:** 10.1111/ped.70373

**Published:** 2026-03-16

**Authors:** Mirei Sasai, Yuri Kataoka, Sakina Kuge, Kana Ogino, Yuhei Konishi, Yutaka Takemura, Keisuke Sugimoto

**Affiliations:** ^1^ Department of Pediatrics Kinki University Faculty of Medicine Sakai‐shi Osaka Japan

**Keywords:** anoscopy, eosinophilic proctitis, FPIES, neonatal, non‐EoE EGID

A female neonate born at 38 weeks and 5 days gestation, weighing 2614 g (−1.24 SD) and measuring 47.9 cm (−0.37 SD) in length, with no notable perinatal history. The first feeding began a few hours after birth. Bloody stool was recognized approximately 24 h later. A shallow skin desquamation‐like anal fissure was observed at the 12 o'clock position. Vomiting appeared from 2 days of age. She was transferred to our hospital for further examination and treatment.

On admission, a blood test showed a mild inflammatory response and peripheral eosinophilia. Both newborn and expanded newborn screening results were negative. An abdominal ultrasound, upper gastrointestinal series, and barium enema revealed no anatomical abnormalities and wall thickening of the digestive tract. An antigen‐specific lymphocyte proliferation test (ALPT) showed no specific finding.

Anal fissure healed spontaneously by 4 days of age, but the bloody stool continued. In addition, poor feeding appeared from 5 days of age. We gradually removed the formula milk and mother's milk, but the symptoms persisted. From day of age 13, we ordered nil per os (NPO), and the symptoms disappeared. When enteral nutrition was gradually resumed from 16 days of age, there was an increase in CRP and eosinophil ratio. At 30 days of age, a rectal biopsy was performed using an anoscope. Within the scope of the anoscope (from the rectum to the anal canal), no abnormalities such as redness or erosion were observed on the mucosal surface. However, in the collected samples, some edema‐like changes and small areas of bleeding were observed in part of the mucosal lamina propria, along with localized eosinophilic infiltration (60–90 cells/HPF) (Figure 1). Histologically, we diagnosed it with eosinophilic proctitis.

**FIGURE 1:**
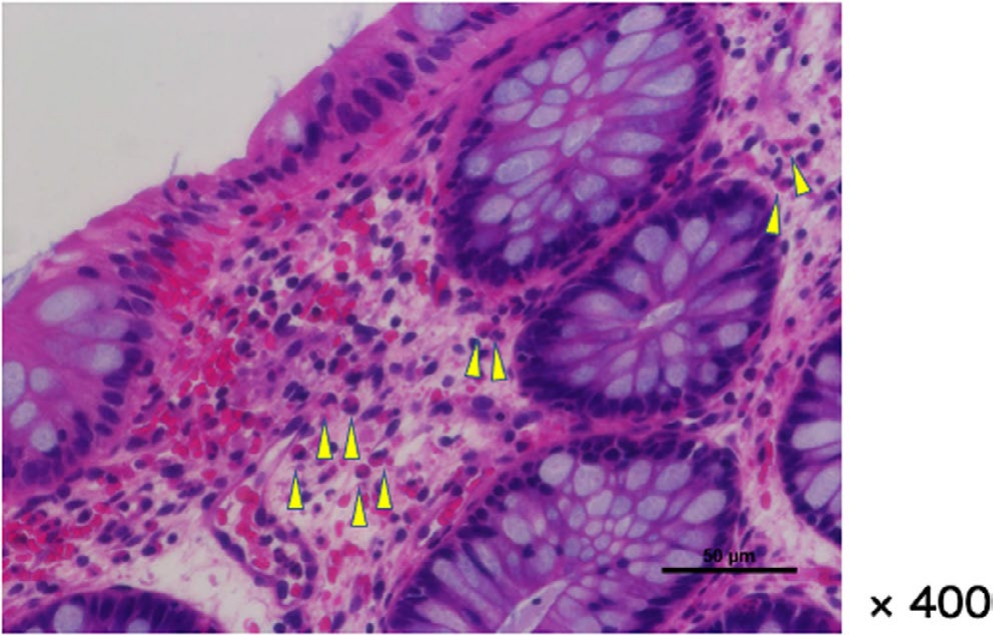
Biopsy specimen of the rectal mucosa (hematoxylin and eosin stain). Rectal mucosa samples were collected using biopsy forceps (2.0 gastric mucosa biopsy forceps) at two points each at 3 cm and 2 cm from the anal margin, and one point at 1 cm. In the collected samples, some edema‐like changes and small areas of bleeding were observed in part of the mucosal lamina propria, along with localized eosinophilic infiltration (60–90 cells/HPF). Mild inflammatory cell infiltration, mainly consisting of lymphocytes and plasma cells, was observed around it.

In addition to bloody stools, this case also presented with vomiting symptoms, leading us to strongly suspect non‐esophageal eosinophilic gastrointestinal disease (non‐EoE EGID).^1^ Symptoms and inflammatory findings were difficult to control with food elimination and elemental diets, so prednisolone (2 mg/kg/day) was started at 30 days of age. Enteral nutrition was resumed at 38 days of age. While carefully monitoring clinical symptoms and laboratory data, the enteral nutrition formula was adjusted and PSL was gradually tapered off (Figure S1). Regular follow‐up was conducted even after discharge. There has been no recurrence even after introduction of weaning foods.

Food protein‐induced enteropathy is a known cause of bloody stools and vomiting in neonates. In recent years, there has been an increase in cases showing eosinophilic infiltration similar to EGID among non‐IgE‐mediated gastrointestinal food allergies (non‐IgE GIFA), including Food Protein‐Induced Enterocolitis (FPIES).^2^

In this case, we performed a rectal biopsy and, based on the pathological findings, diagnosed non‐EoE EGID and administered systemic steroid treatment, which resulted in improvement of symptoms and blood test data.

In EGID, serum CRP levels at onset may reflect the severity of intestinal inflammation.^3^, ^4^ In this case, serum CRP levels are considered a relatively simple and useful test for evaluating intestinal inflammation, as well as for assessing peripheral blood eosinophil counts.

Food Protein‐Induced Allergic proctocolitis (FPIAP) is pathologically diagnosed as infantile eosinophilic colitis. There are case reports of chronic FPIES that do not show acute symptoms in the oral food challenge (OFC) test.^2^ This case is likely chronic FPIES, with a possible diagnosis of non‐IgE‐mediated gastrointestinal food allergy overlapping with FPIAP and EGIDs.

There are also reports stating that “approximately 60% of patients with systemic steroid therapy for non‐EoE EGID experience recurrence,”^2^ After carefully weighing the risks and benefits,^5^ we would like to consider colonoscopy to examine the entire colon when symptoms recur.

## AUTHOR CONTRIBUTIONS

MS managed the patient and wrote the manuscript. Yuri Kataoka managed the patient and assisted in drafting the manuscript. SK, KO, Yuhei Konishi, and YT advised on patient management and treatment. KS performed critical review and revision. All authors have read and approved the final version of manuscript.

## INFORMED CONSENT

Written informed consent was obtained from the patient's parents for their anonymized information to be published in this article.

## CONFLICT OF INTEREST STATEMENT

The authors declare no conflict of interest.

## Supporting information


**Figure S1.** Posthospitalized progress. The horizontal axis shows days of age. The types of enteral nutrition are shown in light gray boxes. Details are described later. (a) Mother’s milk or Formula milk (from 0 to 4 days of age). (b) Mother’s milk or 16.0% Elemental formula (from 5 to 12 days of age). (c) NPO (from 13 to 15 days of age). (d) 10% glucose solution (from 16 days of age). (e) Electrolyte supplement (from 17 to 37 days of age). (f) 8.0% Elemental formula (from 38 to 43 days of age). (g) 10.7% Elemental formula (from 44 to 50 days of age). (h) 13.3% Elemental formula (from 51 to 57 days of age). (i) 16.6% Elemental formula (from 58 to 71 days of age). (j) Formula milk (from 72 days of age). The period during which bloody stools were observed is indicated by the hatched box. Steroid therapy was initiated at 2 mg/kg/day starting on 30 days of age. The steroid dose was gradually tapered: to 1.6 mg/kg/day starting on 41 days of age, to 0.8 mg/kg/day starting on 48 days of age, and to 0.4 mg/kg/day starting on 55 days of age. Treatment was discontinued on 62 days of age. The graph shows CRP as a solid line and the eosinophil ratio as a dotted broken line for blood tests. The horizontal axis represents the day of age, the left vertical axis shows CRP values, and the right vertical axis shows the eosinophil ratio.

## Data Availability

The data that support the findings of this study are openly available in DOI at [DOI], reference number https://doi.org/10.1067/mpd.2002.127663, https://doi.org/10.3345/cep.2022.01053, https://doi.org/10.1016/0002‐9343(79)90652‐1, https://doi.org/10.3389/fped.2021.642342, https://doi.org/10.5223/pghn.2015.18.4.253.
