# Supplementary figures and images for: A neonatal case of non‐eosinophilic esophagitis type eosinophilic gastrointestinal disease diagnosed by rectal biopsy
Source: Pediatr Int. 2026 Mar 16;68(1):e70373. doi: 10.1111/ped.70373 (PMC12993095; doi:10.1111/ped.70373)

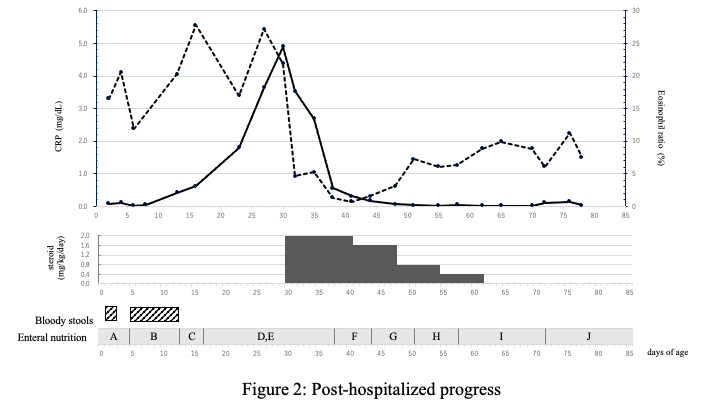

Supplement: Supplementary file 1 — Figure S1. Posthospitalized progress. The horizontal axis shows days of age. The types of enteral nutrition are shown in light gray boxes. Details are described later. (a) Mother’s milk or Formula milk (from 0 to 4 days of age). (b) Mother’s milk or 16.0% Elemental formula (from 5 to 12 days of age). (c) NPO (from 13 to 15 days of age). (d) 10% glucose solution (from 16 days of age). (e) Electrolyte supplement (from 17 to 37 days of age). (f) 8.0% Elemental formula (from 38 to 43 days of age). (g) 10.7% Elemental formula (from 44 to 50 days of age). (h) 13.3% Elemental formula (from 51 to 57 days of age). (i) 16.6% Elemental formula (from 58 to 71 days of age). (j) Formula milk (from 72 days of age). The period during which bloody stools were observed is indicated by the hatched box. Steroid therapy was initiated at 2 mg/kg/day starting on 30 days of age. The steroid dose was gradually tapered: to 1.6 mg/kg/day starting on 41 days of age, to 0.8 mg/kg/day starting on 48 days of age, and to 0.4 mg/kg/day starting on 55 days of age. Treatment was discontinued on 62 days of age. The graph shows CRP as a solid line and the eosinophil ratio as a dotted broken line for blood tests. The horizontal axis represents the day of age, the left vertical axis shows CRP values, and the right vertical axis shows the eosinophil ratio. [file PED-68-e70373-s001.tiff]
